# Formation and Biological Characteristics Analysis of Artificial Gynogenetic WuLi Carp Induced by Inactivated Sperm of Megalobrama Amblycephala

**DOI:** 10.3390/biology14080994

**Published:** 2025-08-04

**Authors:** Xiaowei Xu, Enkui Hu, Qian Xiao, Xu Huang, Chongqing Wang, Xidan Xu, Kun Zhang, Yue Zhou, Jinhai Bai, Zhengkun Liu, Yuchen Jiang, Yan Tang, Xinyi Deng, Siyang Li, Wanjing Peng, Ling Xiong, Yuhan Yang, Zeyang Li, Ming Ma, Qinbo Qin, Shaojun Liu

**Affiliations:** 1 Engineering Research Center of Polyploid Fish Reproduction and Breeding of the State Education Ministry, College of Life Sciences, Hunan Normal University, Changsha 410081, China; xgx1921192165@163.com (X.X.); 17680540702@163.com (E.H.); xiaoqian_0225@163.com (Q.X.); xiuhuang1993@163.com (X.H.) wcq@hunnu.edu.cn (C.W.) 15377324945@163.com (X.X.) 15034676496@163.com (K.Z.) zhouyue202705@126.com (Y.Z.) b13807253562@outlook.com (J.B.) l1530697267@outlook.com (Z.L.) jyc990213@163.com (Y.J.) 18311508595@163.com (Y.T.) q2819733767@163.com (X.D.) lsy430181@126.com (S.L.) 19173996279@163.com (W.P.) xiongling@hunnu.edu.cn (L.X.) ayyhmf@126.com (Y.Y.) 15310949170@163.com (Z.L.); lsj@hunnu.edu.cn (S.L.); 2 Yuelushan Laboratory, Changsha 410128, China; 3 Key Laboratory of Chemical Biology and Traditional Chinese Medicine Research (Ministry of Education), College of Chemistry and Chemical Engineering, Hunan Normal University, Changsha 410081, China; mingma@hunnu.edu.cn

**Keywords:** artificial gynogenesis, allosperm effect, *Megalobrama amblycephala*, micro-hybrid, WuLi carp

## Abstract

Artificial gynogenesis is an essential technique for aquaculture breeding. Fertile offspring of the WuLi carp (*Cyprinus carpio* var. Quanzhounensis, 2*n* = 100, WLC) were successfully produced via gynogenesis using ultraviolet-irradiated sperm from the blunt snout bream (*Megalobrama amblycephala*, 2*n* = 48, BSB). As anticipated, gonadal section examination confirmed that all gynogenetic WuLi carp (2*n* = 100, GWB) were female. To investigate whether paternal DNA fragments from BSB were integrated into the GWB genome, comparative analyses of morphological traits, DNA content, chromosomal numbers, 5S rDNA sequences, microsatellite DNA markers, fluorescence in situ hybridization (FISH), growth performance and nutritional composition were systematically conducted between GWB and maternal WLC. The results revealed pronounced maternal inheritance patterns across morphological characteristics, DNA quantification, chromosomal configurations, 5S rDNA sequences and FISH signals, while microsatellite detection unequivocally confirmed paternal BSB DNA fragment integration into the GWB genome. Remarkably, GWB demonstrated significantly superior growth performance and elevated unsaturated fatty acid content relative to the maternal line. This approach not only addressed germplasm degradation in WLC but also provided valuable theoretical foundations for breeding programs in this commercially significant species.

## Simple Summary

This study successfully produced all-female offspring (GWB, 2*n* = 100) of WuLi carp (*Cyprinus carpio* var. Quanzhounensis, 2*n* = 100, WLC) via artificial gynogenesis using ultraviolet-inactivated sperm of blunt snout bream (*Megalobrama amblycephala*, 2*n* = 48, BSB). GWB inherited most traits from the maternal line, including morphology, DNA content, chromosome structure, and key gene sequences. However, molecular marker detection confirmed that BSB DNA fragments were successfully integrated into GWB’s genome. Additionally, GWB showed faster growth and higher unsaturated fatty acid content than the maternal WLC. These results provide theoretical support for breeding new WLC germplasm with faster growth and higher nutritional value, holding great significance for aquaculture.

## 1. Introduction

The artificial induction technology of gynogenesis is an important technique in aquaculture breeding. It stimulates egg development and the doubling of genetic material by using allosperm, thereby producing all-female offspring. Two main steps are involved in this technology: the activation effect of allosperm and the doubling of the genetic material of eggs [1,2]. The aquaculture industry mainly uses artificial gynogenesis technology to rapidly establish pure lines, utilize unisexual populations, and determine sex genetic mechanisms [3,4,5]. Artificial gynogenesis technology has a very high practical value and holds great significance in the field of aquatic animal breeding. For instance, there have been relevant reports on the successful application of artificial gynogenesis technology in fish species such as grass carp *(Ctenopharyngodon idella*, 2*n* = 48) [6] and mandarin fish (*Siniperca chuatsi*, 2*n* = 48) [7]. However, there has been no pertinent research on the gynogenesis of WuLi carp *(Cyprinus carpio* var. Quanzhounensis, 2*n* = 100, WLC) (Hehua carp).

The WuLi carp *(Cyprinus carpio* var. Quanzhounensis, 2*n* = 100, WLC) is an indigenous fish unique to China and has a long aquaculture history [8]. It earns its name because it has long been found in paddy fields and feeds on falling rice flowers. It is also known as the “Hehua carp” and belongs to the Cyprinidae family of warm-water fish. It has a sturdy body shape, a slightly purple (dark brown) coat, a black back, and bright colors. It has an omnivorous diet, grows fast, and has a strong reproductive ability. Moreover, it has delicate meat with few bones and lots of flesh and tastes delicious with a high protein content. The bones are soft, and it does not smell like fish [9,10]. A significant percentage of inbreeding results from breeding methods adopted by certain WLC farmers at present, which use commercial fish without proper selection or direct buying from the market. Therefore, a series of problems emerge, such as germplasm degradation, uneven fry quality, frequent disease occurrence, and delayed growth and development, which become obstacles restricting the development of the WLC industry [11]. Currently, research on WLC mainly focuses on the aspects of paddy field aquaculture [12] and its quality (in terms of nutrition and stress resistance) [13]. In terms of breeding, researchers usually adopt selective breeding or self-crossing methods for reproduction studies on WLC [14]. The problem of germplasm resources is certainly the core issue in the WLC or the broader carp industry that needs to be resolved urgently.

Blunt snout bream (*Megalobrama amblycephala*, 2*n* = 48, BSB) and WLC belong to the Cyprinidae family. BSB exhibits rapid growth and has tender flesh. Notably, the semen quality of male BSB is relatively high, which renders it an optimal stimulant source for the induction of gynogenesis. There is currently a lack of relevant research on the hybridization between WLC and BSB. This study aimed to induce artificial gynogenesis in WLC using inactivated sperm of BSB to obtain gynogenetic WuLi carp (2*n* = 100, GWB) and establish a homozygous line. A comprehensive analysis was performed of the morphological characteristics, DNA content, chromosome number, 5S rDNA sequence, microsatellite DNA sequence, and fluorescence in situ hybridization (FISH) results of the GWB. To verify the genetic origin of the offspring, we compared their genetic characteristics to those of their parents to confirm that the key genetic materials of GWB had originated from the maternal parent, with only a small amount of insertion from the paternal parent. For instance, we utilized fluorescence in situ hybridization (FISH) experiments to confirm that GWB had been mainly inherited from the maternal parent, and the technique has often been employed to clarify parental–offspring relationships [15]. Additionally, microsatellite DNA could also have been used to detect the integration of paternal fragments into the offspring [16].

## 2. Materials and Methods

### 2.1. Ethics Statement

All experimental fish used in this study were provided by the Hunan Normal University. The researchers strictly followed ethical guidelines for laboratory animals (Ethics Approval Document No. 380 (2025) from the Biomedical Research Ethics Committee of Hunan Normal University (Ethics Review Section)). The experimental fish were supplied with adequate nourishment and were maintained at specific pH (7.0–8.5), water temperatures (22–24 °C), and dissolved oxygen levels (5.0–8.0 mg/L). For humane handling before necropsy, the selected fish were anesthetized with 100 mg/L MS-222 (procured from Sigma-Aldrich, St. Louis, MO, USA).

### 2.2. Technical Method for Inducing Gynogenesis in WuLi Carp

To induce the gynogenesis of WLC, the cold-shock method was adopted to suppress the release of the second polar body of the egg cell. The specific procedures used were as follows: 2-year-old BSB was used as the donor of heterologous sperm. After collecting the 5 mL sperm, it was diluted with Hank’s (Sangon Shanghai) solution at a ratio ranging from 10 times. Approximately 3 mL of the diluted sperm was carefully and evenly spread over the bottom of a 25 mL Petri dish, and the remaining diluted sperm was stored in a refrigerator at 4 °C for later use.

The Petri dish was placed on an ice plate wrapped in a dry towel. The sperm in the dish was inactivated by irradiating it with a 15 W ultraviolet lamp placed at a distance of about 30 cm. Meanwhile, the whole setup was covered with a light-shielding cloth to prevent photoreactivation of the sperm. During 12 min of ultraviolet irradiation process, the Petri dish was manually shaken every 1–3 min, and the sperm motility was monitored under a microscope. The irradiation was terminated when about 50% of the sperm lost their activity. After the irradiation, the Petri dish was placed sideways in a dark place to allow the inactivated sperm to accumulate on one side. Then, the aggregated sperm was aspirated with a syringe, transferred into a light-proof centrifuge tube, and stored at 4 °C.

When the 2-year-old female WLC (Wuli Carp) had completed ovulation, the eggs were carefully squeezed into a ceramic basin, and the inactivated heterologous sperm was poured into the basin and uniformly stirred. The fertilized eggs were then evenly dispersed in a Petri dish with constant-temperature water that had been pre-heated to 23–25 °C, with enough space maintained between each fertilized egg to ensure sufficient oxygen supply during the incubation process. The culture solution in the Petri dish was drained 2 min after fertilization and replaced with ice-cold water at 4 °C. Subsequently, the Petri dish was transferred to a refrigerator at 4 °C for 23 min of cold treatment to inhibit the release of the second polar body and promote the doubling of the genetic material of the eggs. The Petri dish was then quickly removed from the refrigerator, and the ice-cold water was drained out and replaced with 23 °C room-temperature water to allow the fertilized eggs to continue their normal development. Under each set of conditions, 1000 fish eggs were randomly selected, and their hatching rates were statistically analyzed.

### 2.3. Measurement of Countable Traits

In this study, 90 experimental fish, including 30 female WLC, 30 male BSB, and 30 female GWB, were randomly selected for detailed morphological feature observations. The goal was to identify the morphological differences between the parent fish and their hybrid offspring, thus providing a solid data foundation for further genetic and breeding studies. On the one hand, the measurable traits of the experimental fish were measured, including standard length (SL), total length (WL), body depth (BH), head length (HL), head depth (HD), caudal peduncle length (CPL), and caudal peduncle depth (CPH). It was possible to understand the overall morphology and body proportions of the fish using these data. Additionally, a comprehensive assessment of the morphological features was also conducted to accurately calculate the ratios of WL/SL, BH/SL, HL/SL, HD/SL, CPL/SL, and CPH/SL. These ratio data are of great significance for understanding the adaptability and biological characteristics of the fish. Conversely, the countable traits of the experimental fish, such as the number of lateral line scales, scales above and below the lateral line, dorsal, pectoral, pelvic, and anal fin rays, were observed and counted. As different fish species often vary in the numbers of fin rays and scales, this procedure helps identify fish species. All data obtained throughout the experiment were subjected to a one-way analysis of variance using Statistical Package for the Social Sciences software (version 17.0) to clarify the differences among different experimental groups. Moreover, Duncan’s test was used for multiple comparisons. The experimental results are presented in the form of “mean ± standard error”, and the significance level was set at *p* < 0.05.

### 2.4. Study on the Ploidy of Gynogenetic Offspring

In the study, to elucidate the ploidy status of the GWB, 30 experimental fish were selected from both the parental and the offspring generations. In this experiment, a flow cytometer (Partec Company, München, Germany) was used to assess the DNA content of their blood cells and determine the number of chromosomes by preparing chromosome sections.

The protocol for detecting DNA content refers to the method described in a previous study [7]. Sterile syringes and anticoagulant were prepared, with each syringe pre-filled with 0.2 mL of anticoagulant. The anticoagulant was used to prevent blood clotting, ensuring that the blood samples remained in a liquid state during the experiment for subsequent analysis. Blood samples were collected from the caudal vein of the fish to minimize harm to the specimens. After collection, the samples were immediately stored in an icebox to maintain freshness and cellular viability. For nuclear staining, sterile EP tubes were prepared, each containing 0.3 mL of DAPI (Nuclei extraction solution, produced by Partec GmbH, Görlitz, Germany) staining solution and 1 mL of 8% physiological saline. DAPI, a fluorescent dye, specifically binds to DNA, allowing visualization of nuclear morphology under a microscope. Using a 1 μL micropipette, the collected blood was gradually added to the DAPI-containing EP tubes until the solution turned light red. This step ensured thorough mixing of the blood sample with the staining solution. After sample preparation, the tubes were kept in the dark for 10–15 min to allow sufficient DAPI penetration into the nuclei. Following the incubation period, the samples were filtered through a 20 μm mesh filter to remove unbound dye and cellular debris, then diluted for flow cytometry analysis. The DNA content of WLC was used as a calibration control.

The experimental protocol for chromosome section preparation also refers to the method described before [17]. On Day 1 at 8:30 PM, fish were injected with 4 mg/mL PHA (Roche Life Science Products, Basel, Switzerland) (10 mg/kg body weight; volume = 0.0025 × weight) near the pectoral fin. On Day 2 at 8:30 AM, a second PHA injection (15 mg/kg; 0.00375 × weight) was administered. Three hours later, a third PHA injection (6 mg/kg; 0.0015 × weight) and colchicine (2.5 mg/mL; 4 mg/kg; 0.00075 × weight) were injected on the opposite side. After 1 h, fish were sacrificed. Kidneys were aseptically extracted via dissection from the cloaca, rinsed with physiological saline, minced into a homogenate in a Petri dish, and transferred to a 15 mL EP tube. The homogenate was diluted to 4 mL with saline, pipetted vigorously (~200×), adjusted to 12 mL, and repipetted. After 10 min of sedimentation, the supernatant was transferred to a new tube. Then, 4 mL of KCl was added, mixed, diluted to 10 mL, incubated for 60 min (sediment removed every 10 min), and centrifuged (1500 rpm, 5 min). The pellet was fixed three times with Carnoy’s fixative (methanol:glacial acetic acid = 1:3; 2 mL added initially and then 6 mL, mixed, held for 15 min, centrifuged at 1500 rpm for 5 min per cycle). The final pellet was stored in fixative at 4 °C. Fixed cells were dropped onto pre-chilled slides (−20 °C, 24 h), flame-dried, stained for 45 min with Giemsa solution (Na_2_HPO_4_ + NaH_2_PO_4_ + Giemsa) (Sangon Shanghai), rinsed gently, air-dried, and examined microscopically.

### 2.5. Study on the Fertility of Gynogenetic Offspring

The gonad structure of 30 GWB (exclusively female progeny) was observed using gonadal paraffin sectioning to examine their fertility. The specific experimental methods have been described in previous publications [17]. The gonadal structures of female parents of the same age were used as a control. Paraffin-embedded gonadal sections were prepared for GWB at 6 and 24 months of age to assess gonadal development. Detailed experimental protocols followed previously described methods [17]. The specific procedures were as follows: Immediately after capture, fish were sterilized and dissected to extract gonadal tissue, which was fixed in Bouin’s (Sangon Shanghai) solution (refreshed once during 1–3 days) followed by storage in 70% ethanol. The next day at 8:00 AM, tissues underwent sequential dehydration through graded ethanols (70%→100%, 1 hr per concentration), followed by clearing in xylene-ethanol (1:1, 45 min) and pure xylene (≤ 15 min, repeated until transparent). One hour prior, a 60 °C oven was pre-heated to melt paraffin wax; tissues were infiltrated in molten paraffin (60 °C, 2 h), embedded in molds, and cooled to solid blocks. Blocks were trimmed into cubes, sectioned at 5 μm using a microtome, floated on 42 °C water, and mounted on glycerol-coated slides for drying (42 °C, 48 h). Slides were dewaxed in xylene (6 h), rehydrated through descending ethanols (100% → 70%, 5 min per step), stained with hematoxylin (45 min), rinsed, differentiated in 0.5% HCl (2 s) and 0.2% NaOH (10 s), counterstained with eosin (1 min), dehydrated in ascending ethanols, cleared in xylene (5 min), and coverslipped with glycerol resin under bubble-free conditions before air-drying for microscopic evaluation.

### 2.6. 5S rDNA Detection Experiment

A total of 10 female parents, 10 male parents, and 10 GWB were selected for the genetic analysis experiment. First, the caudal fins of the fish were thoroughly washed with a 70% alcohol solution to remove any potential microorganisms and impurities. Once the washing was completed, autoclaved scissors were used to cut off the caudal fins. These caudal fins were immediately placed into pre-labeled EP tubes, and then DNA was extracted using the Omega tissue DNA kit. Polymerase chain reaction (PCR) amplification was carried out using 5S rDNA primers (forward: GCTATGCCCGATCTCGTCTGA; reverse: CAGGTTGGTATGGCCGTAAGC). The PCR reaction system included 15 μL of 2 × Rapid Taq Master Mix (Sangon Shanghai), 1.5 μL of forward primer (Sangon Shanghai), 1.5 μL of reverse primer (Sangon Shanghai), 3 μL of DNA, and 9 μL of TE buffer. The PCR reaction procedure was as follows: pre-denaturation at 95 °C for 5 min; 32 cycles of denaturation at 95 °C for 45 s, annealing at 50 °C for 45 s, and extension at 72 °C for 30 s; final extension at 72 °C for 10 min; and storage at 4 °C. The primers were designed using NCBI.

### 2.7. Fluorescence in Situ Hybridization (FISH) Experiment

In this experiment, the chromosome fixative prepared in the chromosome experiment was used. After the normal dropping of the slides, they were baked in an oven at 70 °C. Meanwhile, the water bath was pre-heated in advance (70, 80 °C). The 188 bp sequence in the 5S rDNA of the male parent BSB was used as the probe in this experiment. The probe hybridization solution was prepared before starting the FISH experiment. The purified probe (5 µL), deionized formamide (DDM, 4 µL), 50% dextran sulfate (3 µL), and 20× sodium citrate buffer (SSC, 2 µL) were mixed. The slides containing chromosomes were pre-heated in an oven at 70 °C for 1–2 h and then soaked in 2× SSC solution for 30 min to reduce the background signal. The slides were dehydrated using a gradient of 70% and 100% ethanol for 5 min each time and then soaked in 70% 2× SSC/DDM solution for 2 min. After drying, the denatured hybridization solution was poured onto the slides. After sealing the slides, they were placed in a humid box and incubated overnight at 37 °C. Following incubation, the slides were washed twice in a 2× SSC/DDM solution at 43 °C for 5 min each time and then washed in 2× SSC and 1× SSC successively for 5 min each. After drying, 8 µL of FITC was added, and the slides were incubated for 20–30 min in the dark. After another 15 min of washing and drying, 6 µL of DAPI and a fluorescence quencher were added to enhance nuclear staining and reduce the fluorescence background. Finally, the slides were observed under a fluorescence microscope to evaluate the hybridization effect and the specific binding of the probe.

### 2.8. Microsatellite Experiment

The genetic loci in the 20 parent fish and 10 GWB were amplified by PCR using 82 pairs of microsatellite primers. Only the YP46 primer pair (forward: AGTATAAGTTGAGTGGGTG; reverse: TAAAGGGAAATTCTGGT) fulfilled the specificity requirement. The PCR was carried out following the procedures and conditions documented in a previous study [18]. The amplification program initiated with a 5 min pre-denaturation at 94 °C, progressed through repeated cycles of denaturation (94 °C, 30 s), annealing (52.5 °C, 30 s), and extension (72 °C, 30 s), followed by a final 7 min extension at 72 °C, and ended with indefinite storage at 4 °C. The amplified products were separated using 8% polyacrylamide gel electrophoresis to ensure accurate genotypic identification.

### 2.9. Growth Performance Experiment

The juvenile fish of the female parent were used as the control to evaluate the growth performance of the gynogenetic juvenile fish. Thirty individuals weighing approximately 1 g each were selected from each group and placed in 250 × 250 × 100 cm culture tanks. The two groups of juvenile fish were reared under similar conditions with a continuous supply of fish feed. Each experimental tank housed 50 fish, forming two parallel experimental groups. At the beginning of the experiment, 30 fish were randomly selected from each group, and their weights (±0.1 g) were accurately recorded as the baseline data. Sufficient feed was provided in all the culture tanks to ensure uniform feeding conditions. From June to August 2024, 30 fish were selected from each group every month to measure their body length and weight to monitor their growth performance. The following formula was used to determine the average daily gain (ADG) so that the weight gain was accurately reflected [19]: ADG = (Wf − Wi)/(Df − Di), where Wf = the average final weight; Wi = the average initial weight; Df = the final day of the experiment; and Di = the first day of the experiment.

### 2.10. Nutrient Composition Analysis of GWB

Thirty 6-month-old maternal WLC and gynogenetic GWB specimens were sampled from identical culture conditions, surface-sterilized with 70% ethanol, and dissected aseptically. Abdominal cavities were excised via anal-ventral incision using sterilized scissors, with complete viscera removal followed by saline rinsing to eliminate residual blood.

Proximate composition was quantified in muscle tissue: moisture via 105 °C desiccation to constant weight, crude protein by Kjeldahl method, and crude lipids via Soxhlet extraction. For amino acid profiling, lyophilized muscle (0.03 g) underwent hydrochloric acid hydrolysis (110 °C, 24 h) under N_2_ atmosphere. Hydrolysates were filtered, diluted to 50 mL with ultrapure water, dried under N_2_ at 50 °C, reconstituted in 0.02 N HCl, membrane-filtered (0.22 μm), and analyzed via sodium-exchange chromatography (LA8080 system, ninhydrin detection), quantified against standards (g/100 g).

Lipid extracts from lyophilized muscle were derivatized to FAME: samples saponified with KOH/CH_3_OH (50 °C), methylated with 14% BF_3_-CH_3_OH (50 °C, 30 min), extracted with hexane, and analyzed via GC (Trace1310 ISQ; 50 m × 0.25 mm column; 5 °C/min ramp 140 → 210 °C hold 10 min; injector/detector 250/300 °C; N_2_ carrier 7.8 mL/min). Fatty acids were identified by retention indices and quantified by relative peak area (g/100 g).

Statistical analysis employed one-way ANOVA with Duncan’s test (SPSS 17.0), with data reported as mean ± SEM with significance threshold *p* < 0.05.

## 3. Results

### 3.1. Results of Gynogenetic Black Carp Production

**Figure 1:**
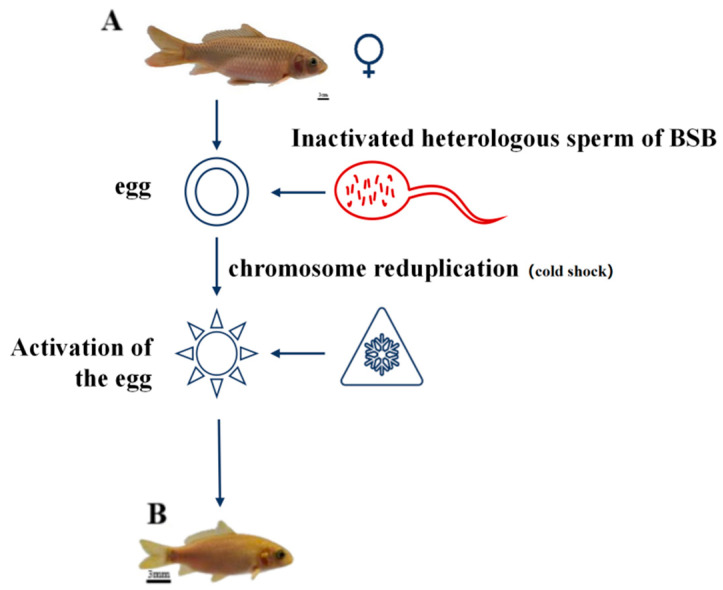
Schematic diagram of artificial gynogenesis in WLC. (**A**): WuLi carp, scale bars: 3 cm; (**B**): Gynogenetic WuLi carp, scale bars: 3 mm.

GWBs of WLC were successfully bred through a meticulously designed artificial gynogenesis breeding experiment. When stimulated by BSB sperm, the GWB exhibited a bluish–yellow body color, as illustrated in Figure 1. These offspring had obvious barbels. Several cold-shock temperatures and durations were set to evaluate the effectiveness of artificial gynogenesis breeding. Finally, it was found that when subjected to a cold-shock temperature of 6 °C for 23 min, the artificial GWB stimulated by BSB sperm achieved the highest hatching rate of 21.7% (Figure 1).

### 3.2. The Measurement Results of Countable Traits

According to the results of this experiment, there were no significant differences (*p* > 0.05) in the number of scales above the lateral line or the number of dorsal fin rays between the GWB and the female parent (WLC), but there were significant differences (*p* < 0.05) when compared to the male parent (BSB). Significant differences (*p* < 0.05) were observed in all the other measurable traits when compared with both parents. The results are displayed in Table 1.

There were no significant differences (*p* > 0.05) in the HD/SL and CPH/SL ratios in the comparison of measurable traits of GWB with those of the parents. Significant differences (*p* ˂ 0.05) were found in the remaining measurable traits compared with the female parent. In terms of WL/SL, there was a significant difference (*p* ˂ 0.05) between GWB and the female parent, but there was no significant difference (*p* > 0.05) when compared to the male parent. The results are summarized in Table 2.

### 3.3. Ploidy Detection Results

**Figure 2:**
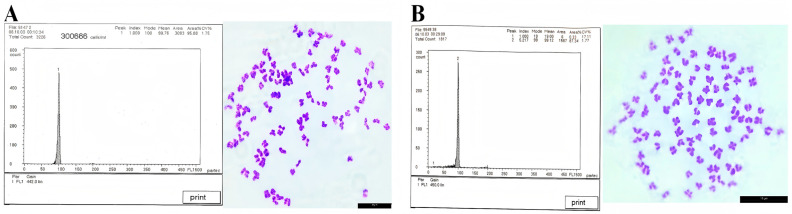
Results of DNA content and chromosomes of WLC and GWB. (**A**): WLC, scale bars: 10 µm; (**B**): GWB, Scale bars: 10 µm.

The blood DNA content of the maternal parent and GWB was comparatively analyzed using flow cytometry analysis. As indicated in Figure 2A,B and Table 3, a thorough data examination revealed that the average DNA content of the GWB was statistically equivalent to that of the maternal parent (*p* > 0.05). Chromosome analysis using prepared chromosome spreads demonstrated that the GWB possessed 100 chromosomes, consistent with diploidy (2*n* = 100) (Figure 2B).

### 3.4. Histological Analysis Results of Gonadal Tissues

**Figure 3:**
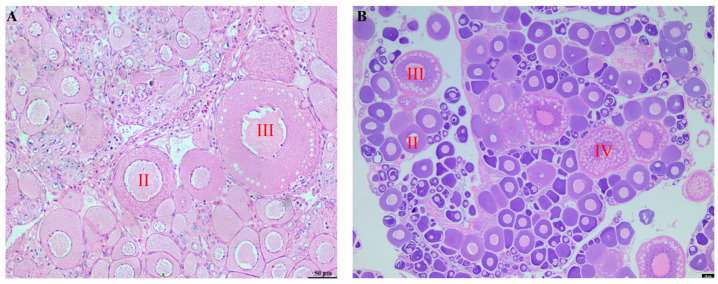
Histology observation of ovaries from GWB. (**A**): 6-month-old GWB, in which oocytes developing into phase II with a minority progressing to phase III, scale bars: 50 µm; (**B**): 24-month-old GWB, in which oocytes of stages II, III, and IV developing in the ovary, scale bars: 50 µm.

Paraffin sectioning of gonadal tissues was employed to characterize the reproductive traits in GWB. The genome-edited fish exhibited slow early gonadal development with a thread-shaped structure. As maternal WLC reach sexual maturity at 2 years, offspring aged 6 and 24 months were analyzed. Histological examination (Figure 3. Histology observation of ovaries from GWB) revealed the following: 6-month-old GWB ovaries were observed under the 40× objective, and extensive oogonial proliferation occurred with most oocytes developing into phase II and a minority progressing to phase III; 24-month-old GWB ovaries were observed under the 20× objective, with oocytes primarily developing into phases II, III, and IV. This well-developed gonadal maturation profile indicates fertility potential in GWB.

### 3.5. 5S rDNA Sequencing Results

**Figure 4:**
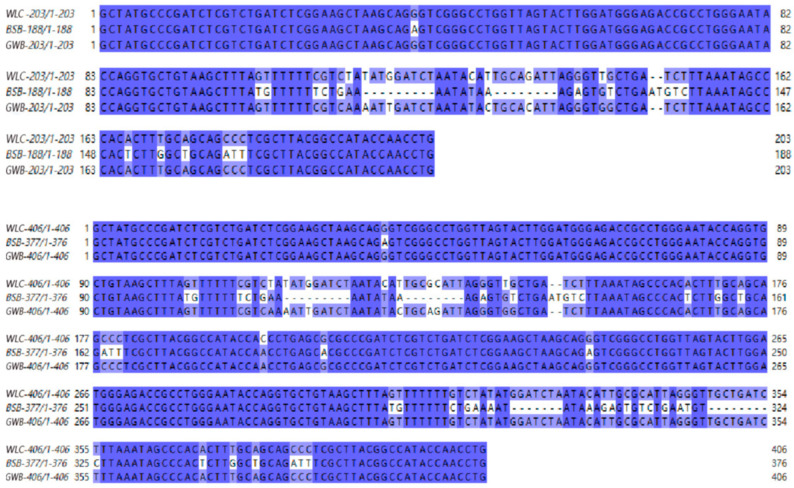
Comparison/alignment of 5S rDNA sequences of GWB and its parents.

Jalview software (https://www.jalview.org/) was used to compare the 5S rDNA sequences of WLC, BSB, and GWB (Figure 4). The results revealed diversity in 5S rDNA monomers among these fish. Specifically, two monomer types were identified in WLC: Type I (203 bp) and Type II (406 bp). Two monomer types, Type I (188 bp) and Type II (376 bp), were observed in BSB. The GWB also exhibited Type I (203 bp) and Type II (406 bp) monomers, similar to the maternal WLC. The sequences were submitted to the NCBI database (PV472174 (WLC 5S rDNA Type I), PV472175 (WLC 5S rDNA Type II), PV472176 (GWB 5S rDNA Type I), PV472177 (GWB 5S rDNA Type II)).

### 3.6. FISH Experimental Results

**Figure 5:**
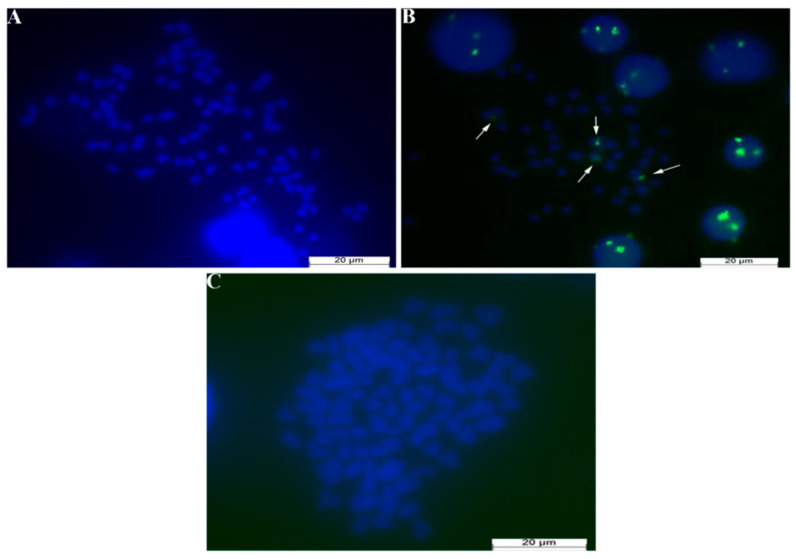
FISH results of cell nucleus from female progeny produced by gynogenesis stimulated by BSB semen. (**A**): WLC FISH results, There are no signal points at all, scale bars: 20 µm; (**B**): BSB FISH results, There are four signal points indicated by white arrows, scale bars: 20 µm; (**C**): GWB FISH results, There are no signal points at all, scale bars: 20 µm.

A 5S rDNA probe specific to BSB was used for detection in the FISH experiment. The maternal WLC displayed no detectable signals in the experimental results (Figure 5A), while the paternal BSB presented four distinct signal spots indicated by white arrows (Figure 5B). The GWB also exhibited no signals in the FISH results (Figure 5C).

### 3.7. Microsatellite Experimental Results

**Figure 6:**
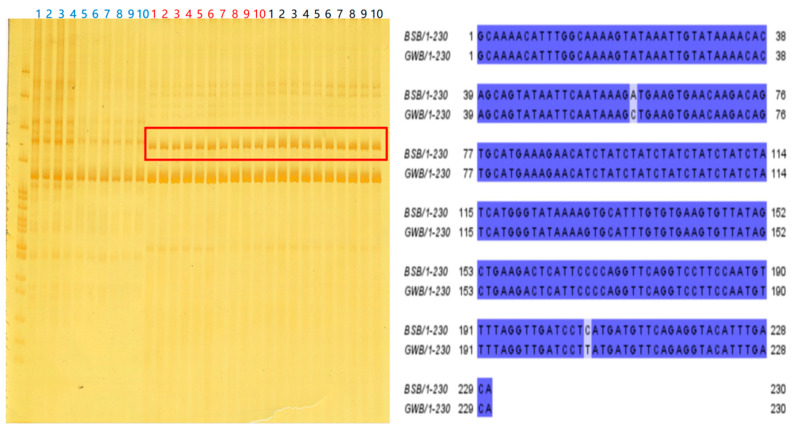
GWB and parental microsatellite results. The numbers in blue font are the female parent WLC, the numbers in red font are the gynogenetic offspring GWB, and the numbers in black font are the male parent BSB. Inside the red frame are the results of the microsatellite testing that were sent for inspection.

The microsatellite analysis experiment was designed to further confirm the genetic origin of the GWB. In this analysis, samples of maternal WLC, paternal BSB, and the GWB were subjected to extensive genetic marker screening using 82 pairs of custom primers. A unique DNA insertion fragment specific to BSB was identified in a specific primer pair (YP46) by meticulous comparison and analysis. This insertion fragment was detected in the paternal BSB and the GWB samples; however, it was absent in the maternal WLC. As illustrated in Figure 6, the paternal BSB-specific insertion fragment (the red square box) was confirmed in the GWB under the specific primers, while this feature was not observed in the maternal WLC.

### 3.8. Growth Performance Experiment Results

A growth performance analysis was conducted based on data from Table 4 to investigate the growth differences between the GWB and the maternal WLC group under the same aquaculture conditions. The GWB group achieved an average body weight of 81.7 ± 2.85 g after 3 months of feeding, while the maternal WLC group demonstrated an average body weight of 70.6 ± 4.28 g. Statistical analysis using Student’s t-test revealed that the average daily gain (ADG) of the GWB and the maternal WLC during the 90-day cultivation experiment was 0.89 and 0.77 g/day, respectively. This significant growth differential suggests that the GWB may exhibit higher growth efficiency or feed conversion ratio compared to the maternal group.

### 3.9. Nutrient Composition Analysis Results

Scaled, headless, and finless muscle samples from 6-month-old WLC and gynogenetic offspring GWB were analyzed for proximate composition, amino acids, and fatty acids (Table 5). Crude protein showed no significant difference between groups. GWB exhibited significantly higher crude lipid content (*p* < 0.05). Among 17 detected amino acids (9 essential, 8 non-essential), glycine was notably elevated in GWB. Fatty acid profiling identified 20 compounds (6 SFAs, 14 UFAs) in GWB versus 19 in WLC (γ-linolenic acid n-6 undetected in WLC). Significantly elevated compounds in GWB included myristic acid (C14:0), pentadecanoic acid (C15:0), heptadecanoic acid (C17:0), α-linolenic acid (C18:3n3), eicosadienoic acid (C20:2), eicosatrienoic acid (C20:3n3), and EPA (C20:5n3), with total UFA content significantly higher than WLC (*p* < 0.05), suggesting enhanced nutritional value.

## 4. Discussion

Gynogenesis is a reproductive strategy of significant biological interest that produces exclusively female offspring. This mode of reproduction results in offspring either being genetically identical to the maternal parent or exhibiting slight variations due to the incorporation of paternal DNA fragments [20]. Aquaculture can benefit from artificial gynogenesis technology in many ways by inducing the production of all-female populations in fish. For instance, females in common carp (*Cyprinus carpio*) typically grow faster than males, enabling all-female populations to achieve higher yields [21]. This approach also allows precise control of sex ratios, minimizes unwanted reproductive behaviors, simplifies farm management, enhances genetic stability [22], and reduces genetic recombination, thereby lowering the risk of inherited diseases [23,24]. Current research on carp gynogenesis spans reproductive biology, genetics, and aquaculture applications, including investigations of genetic regulatory mechanisms during gynogenesis, such as the expression of sex chromosomes, the role of sex-determining genes, and hormone-mediated sex-related pathways [25,26]. Studies also focus on developing methods, including hormonal treatments or biotechnological interventions to induce gynogenesis in carp eggs, and applying these techniques to improve breeding efficiency and select superior varieties [27]. Moreover, the research evaluates the genetic diversity, stability, environmental adaptability, and survival capacity of gynogenetically derived carp populations [2,28].

GWBs were successfully generated through designed artificial gynogenesis experiments. These offspring exhibited a yellowish-green body coloration distinct from the maternal parent, attributed to the stimulation by blunt snout bream (BSB) sperm. Previous studies on artificial gynogenesis in fish have reported phenotypic alterations in GWB [7,29], probably resulting from the allo-sperm stimulation effect. For instance, the number of lateral line scales and dorsal fin rays in GWB differed significantly when compared to the paternal parent. This implies that the inheritance of these traits is maternally dominant. However, other quantitative traits displayed significant differences from both parental lines, potentially due to the insertion of allogeneic sperm-derived DNA fragments, leading to unique morphological variations in GWB [7,8]. Gonadal histology analysis of 24-month-old GWB showed a well-developed structure. Compared to 6-month-old gonads, they contained phase III and IV oocytes, providing evidence that the gynogenetic offspring demonstrated normal fertility.

FISH and 5S rDNA sequence analyses revealed that the GWB exhibited a high genetic similarity to the maternal parent, validating their origin via artificial gynogenesis [30]. However, stimulation by allogeneic sperm led to base substitutions in the NTS region of the 5S rDNA [6]. Microsatellite analysis revealed the insertion of paternal genetic fragments. Notably, growth experiments and nutritional analyses revealed that the GWB exhibited both a higher growth rate and greater unsaturated fatty acid content compared to the maternal WLC. We hypothesize that this phenomenon may be linked to the integration of heterospecific sperm-derived DNA [6,7,8]. Our team proposed the concepts of “macro-hybrid” and “micro-hybrid” based on extensive experimental results in distant hybridization and gynogenesis of fish. A macro-hybrid refers to offspring of distant hybridization that retains two distinct subgenomes derived from each parental species, such as allodiploid and allotetraploid lineages [31,32,33]. In contrast, a micro-hybrid describes offspring generated through artificial gynogenesis, whose genomes are predominantly maternal but incorporate specific paternal DNA fragments, including autodiploid and autotetraploid lines [6,7,34]. The observed genetic diversity in these GWB may provide valuable insights for future breeding programs. Further research should explore the biological impacts of these genetic variations on offspring traits and develop strategies to harness such genetic resources effectively in aquaculture breeding.

## 5. Conclusions

An integrated agricultural system combining rice cultivation and fish farming called rice–fish co-culture features WLC as a unique germplasm resource for Chinese rice–fish systems. Recently, the expanding adoption of rice–fish co-culture has driven increasing demand for high-quality fish germplasm, prompting researchers to explore innovative breeding technologies. This study focused on WLC and used artificial gynogenesis to enable genetic improvement and novel germplasm development for this species. It successfully established GWB populations by inducing ameiotic cleavage in WLC oocytes. This artificial gynogenesis technique effectively controls sex ratios, eliminating reproductive behaviors associated with male individuals during farming. Notably, it was observed that female WLC exhibited faster growth rates and more robust body morphology than males in breeding populations. Accordingly, developing gynogenetic WLC holds significant potential for enhancing aquaculture efficiency and economic returns.

## Figures and Tables

**Table 1:** Comparison of the countable traits among WLC, BSB, and GWB.

Sample	Lateral Line Scales	Scales Above the Lateral Line	Scales Below the Lateral Line	Dorsal Fin	Pectoral Fin	Pelvic Fin	Anal Fin
WLC	35.30 ± 1.14 ^b^	6.00 ± 0.00 ^a^	5.70 ± 0.30 ^a^	III~16–18 ^b^	11–14 ^b^	7–10 ^b^	III~5–7 ^a^
BSB	55.60 ± 1.88 ^c^	11.30 ± 0.60 ^b^	9.40 ± 0.98 ^c^	III~7–9 ^a^	12–15 ^c^	9.00 ^c^	III~25–28 ^c^
GWB	34.52 ± 0.778 ^a^	5.80 ± 0.40 ^a^	6.30 ± 0.40 ^b^	III~15–19 ^b^	9–12 ^a^	7–9 ^a^	III~7–9 ^b^

Note: The capital Roman numerals represent the hard fin rays, and the Arabic numerals represent the soft fin rays. The superscripts a, b, and c indicate differences: if the letters are the same, there is no difference; if the letters are different, there is a difference. Note: The superscripts a, b, and c represent different significant differences, respectively. If the subscripts are the same, there is no significant difference.

**Table 2:** Comparison of the measurable traits among WLC, BSB, and GWB.

Sample	WL/SL	BH/SL	HL/SL	HD/SL	CPL/SL	CPH/SL
WLC	1.32 ± 0.07 ^a^	0.35 ± 0.03 ^a^	0.23 ± 0.01	0.18 ± 0.02 ^b^	0.19 ± 0.02 ^b^	0.13 ± 0.01
BSB	1.24 ± 0.03	0.41 ± 0.01 ^c^	0.21 ± 0.01	0.15 ± 0.01 ^a^	0.14 ± 0.01 ^a^	0.13 ± 0.01
GWB	1.22 ± 0.05	0.38 ± 0.02 ^b^	0.22 ± 0.03	0.20 ± 0.01 ^c^	0.21 ± 0.02 ^c^	0.13 ± 0.01

Note: The superscripts a, b, and c represent different significant differences, respectively. If the subscripts are the same, there is no significant difference. standard length (SL), total length (WL), body depth (BH), head length (HL), head depth (HD), caudal peduncle length (CPL), and caudal peduncle depth (CPH).

**Table 3:** Average DNA contents of WLC and GWB.

Sample	Average DNA	Proportion	
		Observation	Anticipated Value
WLC	99.76	-	-
GWB	99.12	WLC/GWB = 1.01 ^a^	1

Note: a indicates that there is no significant difference between the observed and expected values of the ratio (*p* > 0.05).

**Table 4:** The growth performance data on body weight from WLC and GWB.

Sample	Initial Day (g)	30 Days (g)	60 Days (g)	90 Days (g)
WLC	1 ± 0.23	14.7 ± 2.25	48.5 ± 4.88	70.6 ± 4.28
GWB	1 ± 0.11	26.3 ± 3.63	51.5 ± 5.72	81.7 ± 2.85

**Table 5:** (**A**) WLC and GWB crude protein, crude fat and amino acid statistics. (**B**) WLC and GWB crude fatty acid statistics results.

	(**A**)	
	**WLC (g/100 g)**	**GWB (g/100 g)**
Crude Protein	18.50 ± 0.4582	17.96 ± 0.4932
Crude Fat	3.26 ± 0.1527 ^a^	4.13 ± 0.1527 ^b^
Asp	1.3800 ± 0.0000 ^a^	1.2966 ± 0.0057 ^b^
Thr ^@^	0.6600 ± 0.0000 ^a^	0.6266 ± 0.0057 ^b^
Ser	0.5600 ± 0.0000 ^a^	0.5466 ± 0.0057 ^b^
Glu	1.8166 ± 0.0115 ^a^	1.6666 ± 0.0152 ^b^
Gly	1.1966 ± 0.0057 ^a^	1.2600 ± 0.1000 ^b^
Ala	1.1966 ± 0.0057 ^a^	1.1766 ± 0.0057 ^b^
Cys	0.1000 ± 0.0000	0.1000 ± 0.0000
Val ^@^	0.8033 ± 0.0057 ^a^	0.7366 ± 0.0057 ^b^
Met ^@^	0.2433 ± 0.0057 ^a^	0.2000 ± 0.0000 ^b^
IIe ^@^	0.6933 ± 0.0152 ^a^	0.6400 ± 0.1000 ^b^
Leu ^@^	1.2233 ± 0.0152 ^a^	1.1433 ± 0.0152 ^b^
Tyr ^@^	0.5100 ± 0.0000 ^a^	0.4766 ± 0.0057 ^b^
Phe	0.6566 ± 0.0057 ^a^	0.6066 ± 0.0057 ^b^
Lys ^@^	1.4200 ± 0.0000 ^a^	1.3133 ± 0.0057 ^b^
His ^@^	0.5000 ± 0.0000 ^a^	0.3866 ± 0.0057 ^b^
Arg ^@^	0.9866 ± 0.0057 ^a^	0.9700 ± 0.0000 ^b^
Pro	0.6366 ± 0.0115	0.6366 ± 0.0057
Total Content of Essential Amino Acids	7.0398	6.4930
Total Content of Non-essential Amino Acids	7.5430	7.2896
	(**B**)	
	**WLC (g/100 g)**	**GWB (g/100 g)**
C14:0 *	0.0183 ± 0.0008	0.0241 ± 0.0027
C15:0 *	0.007 ± 0.0005	0.0171 ± 0.001
C16:0 *	0.3501 ± 0.0155	0.3997 ± 0.0136
C16:1 *	0.0642 ± 0.0031	0.0854 ± 0.0049
C17:0 *	0.0087 ± 0.0004	0.0155 ± 0.0006
C18:0	0.1103 ± 0.0012	0.1094 ± 0.0062
C20:2 *	0.0109 ± 0.0007	0.0106 ± 0.0007
C20:3n6 *	0.0193 ± 0.0015	0.0133 ± 0.0008
C20:3n3 *	-	0.0043 ± 0.0005
C22:1n9	0.0195 ± 0.0015	0.0172 ± 0.0015
C20:4n6	0.0474 ± 0.0007	0.0457 ± 0.0032
C20:5n3 *	0.0044 ± 0.0006	0.0070 ± 0.0007
C24:0 *	0.0076 ± 0.0005	0.0128 ± 0.0009
C24:1 *	0.0055 ± 0.0003	0.0040 ± 0.0004
C22:6n3	0.0409 ± 0.0011	0.0403 ± 0.0029
Total Content of Unsaturated Fatty Acids	0.2121	0.2378

Note: The superscript ^ab^ indicates significant difference, and the superscript @ indicates essential amino acids. Asp: Aspartic acid; Thr: Threonine; Ser: Serine; Glu: Glutamic acid; Gly: Glycine; Ala: Alanine; Cys: Cysteine; Val: Valine; Met: Methionine; Ile: Isoleucine; Leu: Leucine; Tyr: Tyrosine; Phe: Phenylalanine; Lys: Lysine; His: Histidine; Arg: Arginine; Pro: Proline. Note: The superscript * indicates significant difference. C14:0-Tetradecanoic acid (Myristic acid); C15:0-Pentadecanoic acid; C16:0—1Hexadecanoic acid (Palmitic acid); C16:1—9-Hexadecenoic acid (Palmitoleic acid); C17:0-Heptadecanoic acid; C18:0—1Octadecanoic acid (Stearic acid); C20:2—11,14-Eicosadienoic acid; C20:3n6—18,11,14-Eicosatrienoic acid (Dihomo-γ-linolenic acid, DGLA); C20:3n3—111,14,17-Eicosatrienoic acid; C22:1n9-13-Docosenoic acid (Erucic acid); C20:4n6—15,8,11,14-Eicosatetraenoic acid (Arachidonic acid, AA); C20:5n3-5,8,11,14,17-Eicosapentaenoic acid (EPA); C24:0—1Tetracosanoic acid (Lignoceric acid); C24:1—115-Tetracosenoic acid (Nervonic acid); C22:6n3—14,7,10,13,16,19-Docosahexaenoic acid (DHA).

## Data Availability

The molecular data generated in this study have been uploaded to NCBI, and the relevant accession numbers are indicated in the Supplementary Materials.

## Supplementary Materials

The following supporting information can be downloaded at: https://www.mdpi.com/article/10.3390/biology14080994/s1, The 5s results of Wuli carp and GWB have been uploaded to NCBI, with the accession numbers PV472174-PV472177. Among them, the paternal BSB 5s sequence compared in the experiment was downloaded from NCBI.
