# Supplementary figures and images for: Formation and Biological Characteristics Analysis of Artificial Gynogenetic WuLi Carp Induced by Inactivated Sperm of Megalobrama Amblycephala
Source: Biology (Basel). 2025 Aug 4;14(8):994. doi: 10.3390/biology14080994 (PMC12383469; doi:10.3390/biology14080994)

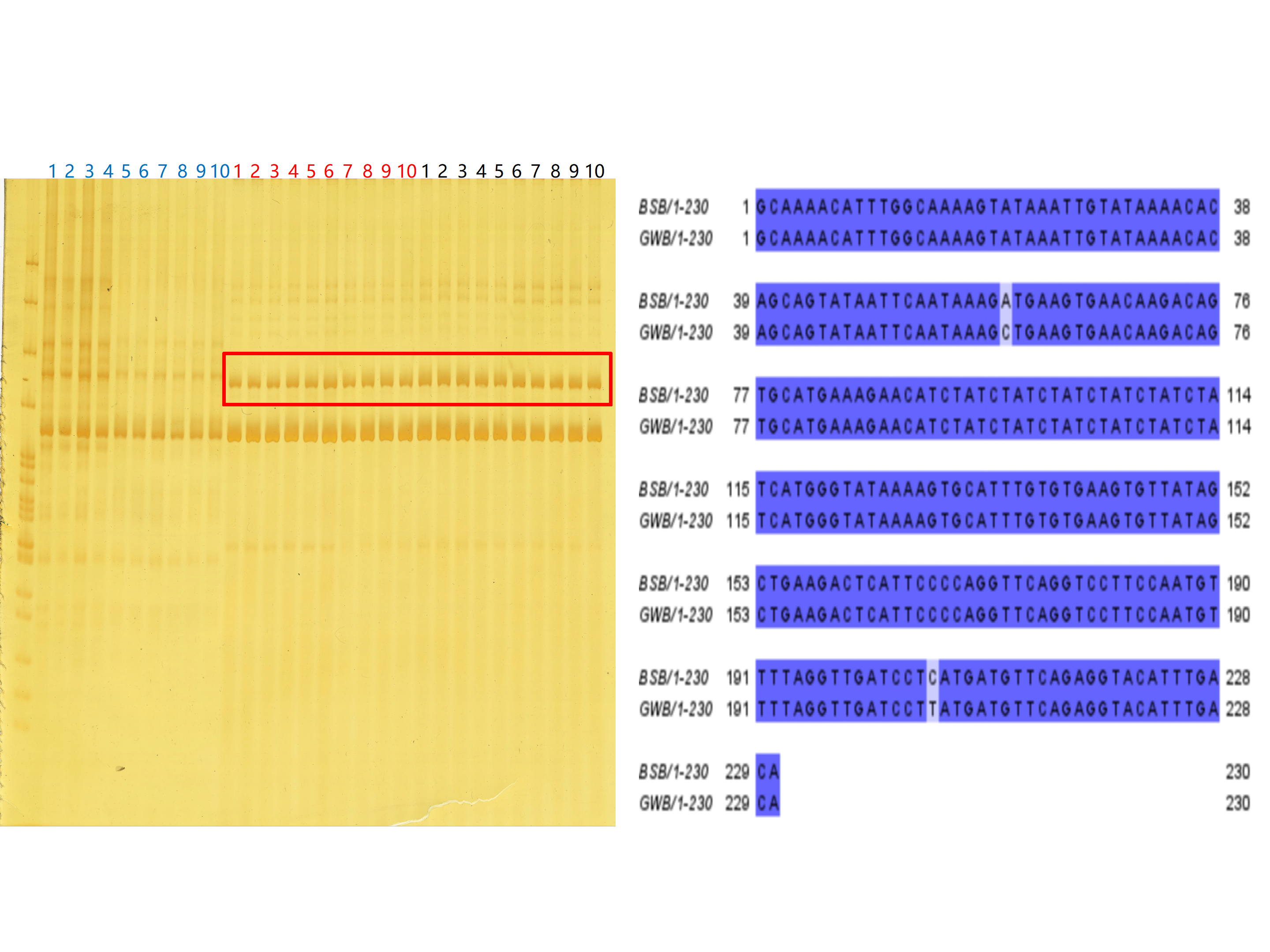

Supplement: Supplementary file 1 [file biology-14-00994-s001.zip › biology-3768903-supplementary.png]
